# Understanding trends in electrochemical carbon dioxide reduction rates

**DOI:** 10.1038/ncomms15438

**Published:** 2017-05-22

**Authors:** Xinyan Liu, Jianping Xiao, Hongjie Peng, Xin Hong, Karen Chan, Jens K. Nørskov

**Affiliations:** 1 SUNCAT Center for Interface Science and Catalysis, SLAC National Accelerator Laboratory, 2575 Sand Hill Road, Menlo Park, California 94025, USA; 2 SUNCAT Center for Interface Science and Catalysis, Department of Chemical Engineering, Stanford University, Stanford, California 94305, USA; 3 Department of Chemical Engineering, Tsinghua University, Beijing 100084, China; 4 Department of Chemistry, Zhejiang University, Hangzhou 310027, China

## Abstract

Electrochemical carbon dioxide reduction to fuels presents one of the great challenges in chemistry. Herein we present an understanding of trends in electrocatalytic activity for carbon dioxide reduction over different metal catalysts that rationalize a number of experimental observations including the selectivity with respect to the competing hydrogen evolution reaction. We also identify two design criteria for more active catalysts. The understanding is based on density functional theory calculations of activation energies for electrochemical carbon monoxide reduction as a basis for an electrochemical kinetic model of the process. We develop scaling relations relating transition state energies to the carbon monoxide adsorption energy and determine the optimal value of this descriptor to be very close to that of copper.

## Abstract (web summary)

Identifying trends in electrocatalytic activity for carbon dioxide reduction can help with catalyst design, but are difficult to define. Here, the authors develop an electrochemical kinetic model of the process, identifying scaling relations relating transition state energies to CO adsorption energy on metal surfaces.

Electrochemical carbon dioxide (CO_2_) reduction to hydrocarbons and alcohols presents one of the great challenges in chemistry. There are known electrode catalysts that can facilitate the process, but they are generally very inefficient—large overpotentials are needed to produce significant reaction rates and the selectivity towards the desired products are often low, with hydrogen evolution being the major competing process^1^,^2^,^3^,^4^. If we could find materials that are able to catalyse this reaction efficiently, we would have a pathway to making fuels and base chemicals in a sustainable way, thus allowing for a zero-emission energy conversion cycle^5^,^6^,^7^,^8^.

Recent experimental reports have focused on the detection of the wide range of carbon-based products on transition metal catalysts^2^, effects of alloying^4^,^9^,^10^, meso- and nano-structuring^3^,^11^,^12^,^13^ and electrolyte engineering^14^,^15^,^16^ on activity and selectivity, as well as *in situ* spectroscopic detection of reaction intermediates^17^,^18^. Theoretical works employing density functional theory (DFT) and various descriptions of the electrochemical interface have usually focused on the mechanism on copper, which is the only pure transition metal capable of reducing CO_2_ to alcohols and hydrocarbons at reasonable faradaic efficiencies^19^,^20^,^21^,^22^,^23^,^24^,^25^. Computational screening for new catalysts has also been attempted based on scaling relations between reaction intermediates, identified using a thermodynamic analysis of the reaction pathway^26^,^27^,^28^. The fact that no catalyst has been found so far that can efficiently catalyse CO_2_ electroreduction to hydrocarbons or alcohols points to a fundamental problem in our current understanding.

In this Article, we present an *ab initio* kinetic model of CO reduction on transition and noble metals that describes trends in catalytic activity and the selectivity of CO reduction over hydrogen evolution. We are primarily interested in CO_2_ reduction to more reduced products than CO, and we therefore focus on CO as the reactant. CO_2_ reduction to CO requires considerably lower overpotentials^29^, and thermodynamic descriptors have been able to accurately predict active catalysts^30^,^31^. We use DFT calculations and an explicit solvent model of the electrochemical interface to estimate potential-dependent activation energies for electrochemical CO reduction. We show that the transition state energy for the H–CO complex scales linearly with the CO adsorption energy for metal surface catalysts, and identify the H–CO versus CO scaling as a crucial determinant of catalytic activity. Model turnover frequencies, polarization curves and selectivity show reasonable agreement with existing experimental data, and suggest stepped sites, such as Cu(*N*11), *N*≥2, to dominate the overall activity compared with Cu(111) and Cu(100) facets. On the basis of the kinetic model, we present two design strategies for more active CO_2_ reduction catalysts.

## Results

### Free energy diagram for CO_2_ reduction to CH_4_

**Figure 1:**
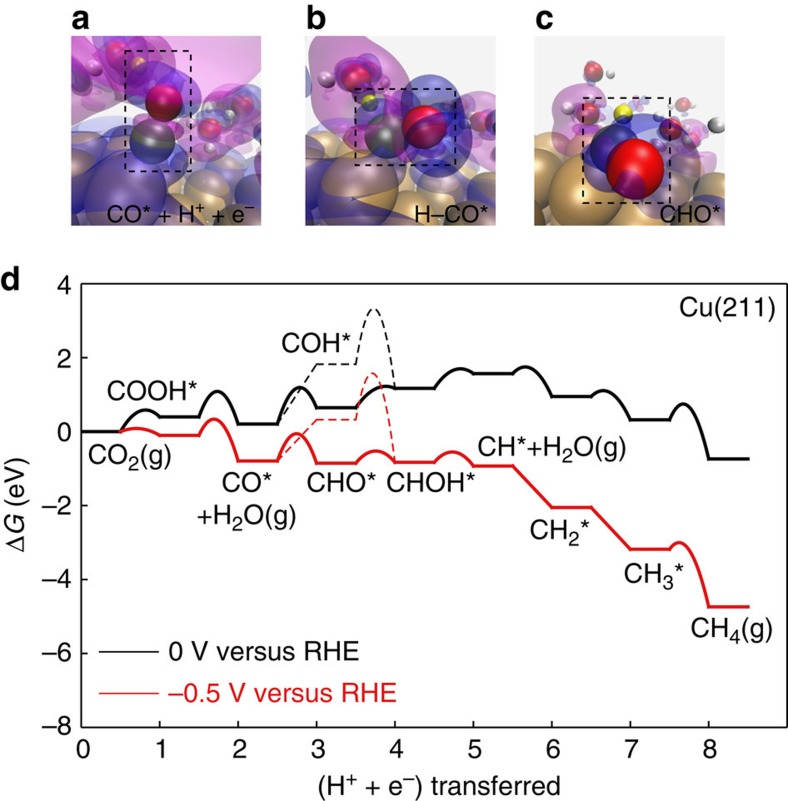
Free energy diagram. (**a**–**c**) charge density difference isosurfaces for the rate determining, proton-electron transfer to *CO to form *CHO. *CO, the *H–CO complex and *CHO have been highlighted with dashed lines. Blue and magenta isosurfaces correspond to charge densities of −0.001 e Bohr^–3^ and +0.001 e Bohr^–3^, respectively. The isosurfaces illustrate the transfer of positive charge to the negatively charged slab along the reaction pathway. (**d**) free energy diagram for the reduction of CO_2_ to CH4 on Cu(211) at 0 V and −0.5 V versus RHE.

Figure 1 shows our calculated free energy diagram including activation free energies for a complete series of elementary steps leading from CO_2_ to CH_4_ (as an example of a possible product) for a stepped Cu(211) surface. We have also included the alternative pathway, CO hydrogenation to COH, which is found to be higher in energy, in contrast to results from simulations using more approximate estimates of electrochemical barriers^20^,^21^. The calculation has five components, which are discussed in more detail in the Methods: (1) we consider coupled electron–proton reaction steps, assuming that the electron transfer happens on a time-scale much faster than the proton transfer. This is a good approximation since the transition state complexes are tightly coupled to the surface, as illustrated by the projected density of states at the transition state (see Supplementary Fig. 6). (2) Reaction energies of all elementary steps are calculated using the computational hydrogen electrode including an explicit solvent layer. All interaction energies are calculated using error estimation ensembles within the BEEF-vdW functional^32^. (3) Activation energies are calculated using an explicit description of the solvent (see Fig. 1a,b for the associated charge density difference isosurfaces). All systems consist of a single layer of hydrogen-bonded water and a 3–4 layer transition metal slab. Excess hydrogens in the water layer charge separate into solvated protons in the Helmholtz plane with countercharge in the slab. Barriers were determined using the climbing-image nudged elastic band method^33^. (4) Free energies *G=E+E*_ZPE_*–*TS are estimated by including zero point energies and entropy contributions calculated in the harmonic approximation^34^. Transition state energies are corrected by the zero point energies of transition state complexes, but no configurational entropies are included in accordance with transition state theory. All corrections are included in Supplementary Table 1. (5) The potential dependence of the activation energies are calculated as described in refs 35, 36. Assuming a standard hydrogen electrode work function of 4.4 eV, activation energies are extrapolated to a work function of 4.0 eV, which corresponds to 0 V versus RHE at pH 7. All activation energies are referenced to the aqueous protons in bulk solution using the computational hydrogen electrode^37^.

Figure 1d shows that on Cu(211) the elementary reaction step involving *CO hydrogenation to *CHO has the highest free energy barrier; the corresponding charge density isosurfaces along the reaction pathway are shown in Fig. 1a–c. We therefore focus on trends in the rate of CO hydrogenation on other metals and surface structures, to understand trends in CO_2_ reduction activity. Catalysts far from Cu in the periodic table may have larger activation energies for other elementary steps, in which case CO hydrogenation will not be the limiting step and the calculated rate will be an upper bound to the rate. We show, in agreement with experiment, that the optimum catalyst is close to Cu^2^, which suggests that we describe the region around the optimum well by concentrating on the CO reduction step.

### Transition state scaling relations

**Figure 2:**
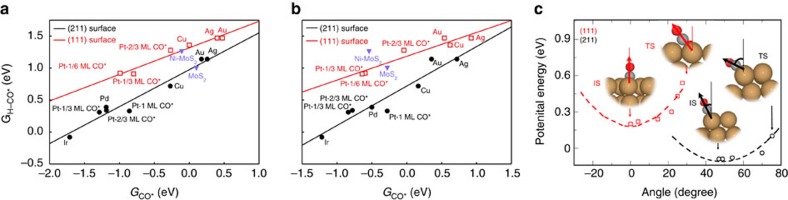
Scaling relations of CO reduction transition state energies on (111) and (211) transition metal facets. (**a**) G_H–CO*_ versus G_CO*_ (**b**) G_H–CO*_ versus G_CHO*_. (**c**) Potential Energy curves for CO adsorbed Cu(111) and Cu(211) as a function of the angle of rotation. For Cu(111) a rotation of adsorbed CO is more energetically costly from initial state to transition state with respect to the stepped Cu(211) surface, which rationalizes the lower transition state energies for stepped surface. All energies are referenced to solvated protons far from the surface at pH=7, electrons at 0* *V versus RHE and gas phase CO at *T*=300 K, *P*=1 bar.

In Fig. 2, we show the CO reduction transition state free energies at 0 V versus RHE for several metals and coverages, and for two different surface orientations with fcc(111) surfaces representing close-packed facets, and fcc(211) surfaces representing low-coordinated, step-like sites. The transition state energies are plotted as a function of the CO adsorption energy, and there is a clear scaling relation between the two. This is the first identification of a transition state scaling relation for electrochemical CO reduction. The scaling relations are surface structure-dependent, like in thermal surface processes^38^.

### Microkinetic modelling

**Figure 3:**
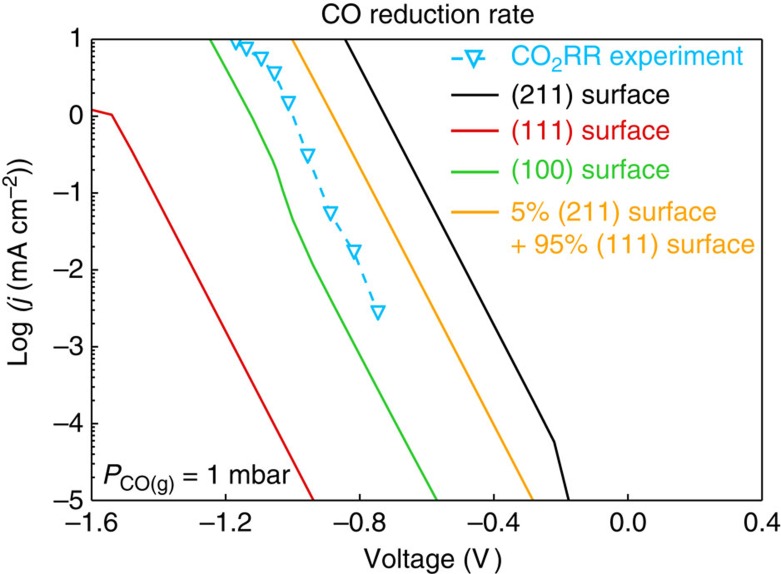
Polarization curves. For Cu(111), (100) and (211) facets at pH=7, 1* *mbar CO_(g)_ for CO reduction to CH_4_. Experimental data is from refs 2, 43 for CO_2_ reduction for all post-CO products, pH=7, where a backpressure of CO of 1* *mbar was estimated^7^. CO_2_RR, carbon dioxide reduction rate.

We then develop a mean field kinetic model to describe the potential-dependent rate of CO reduction to more reduced products. The model includes adsorbate–adsorbate interactions in a self-consistent way^39^ (details in the Supplementary Note 4). The model is devised to describe trends in catalytic activity. Even if absolute rates are not always quantitatively described by DFT calculations, variations in activity amongst a group of catalysts, such as transition metals, are described considerably better^40^. The reason is that the intrinsic error in DFT calculations tends to be systematic in the sense that if one metal over-binds intermediates or transition states, so do the other metals. Having said that, the model describes the variation of current density and selectivity with potential for Cu quite well. Figure 3 shows the theoretical polarization curves for CO reduction for Cu(111) and (211), as well as the experimental CO_2_ reduction curve from ref. 41 for all products further reduced from CO. A CO backpressure of 1 mbar was used. We note that there is a large uncertainty in estimating the effective CO backpressure. But the trend remains unchanged over a range of pressures. See Supplementary Fig. 4 for the pressure dependence of the kinetics. The stepped Cu(211) surface has a significantly higher current density at given potential with respect to Cu(111) and Cu(100) facets. We also include the case where the number of step-like sites are in the range observed on single crystal surfaces, 5% (ref. 44), showing that within the uncertainly of our model (DFT, mean field kinetics and the number of active sites) and of the experiments (active site area and diffusion limitations) our description is quite good, both in terms of the Tafel slope and the absolute rates.

**Figure 4:**
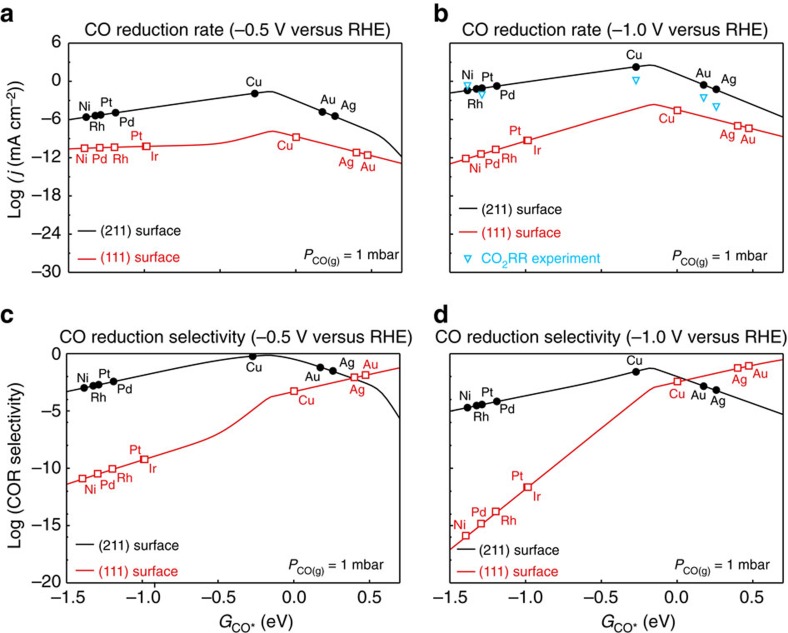
Activity volcanoes for flat (111) and stepped (211) transition metal surfaces as a function of CO binding energy. At (**a**) −0.5* *V versus RHE, (**b**) −1.0* *V versus RHE, as determined through microkinetic modelling. CO_2_ reduction experimental data from refs 2, 41, 43; CO pressure estimated^66^ to be 1 mbar under CO_2_ reduction conditions, pH=7. Selectivity towards CO reduction at (**c**) −0.5 V versus RHE, (**d**) −1.0 V versus RHE, defined as the rate of CO reduction relative to the sum of the rate of CO reduction and hydrogen evolution. CO_2_RR, carbon dioxide reduction rate.

Since the CO adsorption energy defines the activation energies through the scaling relation (Fig. 2a), we can derive the rate of electrochemical CO reduction as a function of the CO adsorption energy for two different surface structures, as shown in Fig. 4. for potentials −0.5 and −1.0 V versus RHE. All CO adsorption energies for the various metals indicated correspond to that calculated at low-coverage, with 1 *CO per 3 × 3 sized unit cell. The fact that the low-coverage CO adsorption energies are used in the volcano plots is merely a convention to determine which energy we use to characterize a given metal. The error bars on the relative rates, derived from BEEF-vdW ensembles^32^, are shown in Supplementary Fig. 4.

Figure 4 shows that according to the model, the stepped (211) surfaces always have a considerably higher activity than the close-packed (111) surfaces for a given CO adsorption energy. The larger catalytic activity of the step-like sites can be traced back to generally lower activation energies (Fig. 2a). These lower activation energies can be rationalized by the accessibility of the C end of *CO to the incoming proton (Fig. 2c), and the ease with which the *CO rotates towards the transition state. Figure 2c shows the potential energy curves for CO adsorbed on Cu(111) and (211) as a function of the angle of rotation from the initial to transition state. On the (211) surface, the overall angle of rotation between the initial state (IS) and transition state (TS) is smaller, and the overall energy change from this rotation is also considerably smaller.

The finding that step-like structures are much more reactive than more close-packed surfaces appears not to be in agreement with experiments on CO reduction on single crystal data^1^,^45^,^46^. The question is what the state of the surface is under reaction conditions. Recent electrochemical scanning tunneling microscopy (STM) imaging show transition metal surfaces to be highly dynamic under electrochemical conditions^47^,^48^, which complicates the direct comparison with experimental single crystal data. We note that recent experiments on polycrystalline copper show oxidation–reduction cycles to give rise to stepped surfaces active for the low-overpotential production of ethanol^47^.

In Fig. 4 and Supplementary Fig. 4, we include experimental CO_2_ reduction data from ref. 2. Our model rationalizes a number of experimentally observed trends in CO reduction rates: Cu is the best elemental metal catalyst, and for the weaker binding metals, the lack of CO coverage limits the rate. On the stronger binding side of the maximum, the variation in rate is smaller, in particular for the step sites. The reason is that the slope of the transition state scaling line is ∼0.6, meaning that the activation energy *E*_a_=*E*_H–CO*_−*E*_CO*_ varies more weakly than the CO adsorption energy from one metal to the next

Apart from the rate of CO/CO_2_ reduction, the selectivity over the hydrogen evolution reaction (HER) is crucial to the efficiency of CO/CO_2_ reduction catalysts. Figure 4c,d shows the selectivity towards CO reduction versus the total current at –0.5 and –1.0V versus RHE. On stepped sites, which should dominate the overall activity, our model suggests that only around Cu in terms of CO adsorption energy is there any substantial selectivity towards CO reduction products, consistent with experimental observations^2^.

### Design strategies for more active catalysts

Figure 4 suggests that Cu is close to the optimum both in terms of rate and selectivity. This hypothesis has not been contradicted by experiments so far. The results suggest two strategies for catalyst design. The first option is to increase the number of step-like sites. We suggest that the high activity forms of nano-structured transition metals that have been reported^3^,^49^,^50^ may in fact result from a larger fraction of steps and edges in these high surface area samples. The importance of special strong-binding sites has been suggested by temperature-programmed desorption of oxide-derived copper^51^. Stabilizing a large fraction of edge and step sites is an important design criterion. As noted above, the dynamic nature of metal surfaces under electrochemical conditions makes it hard to know which sites are on a given surface and even harder to control them.

**Figure 5:**
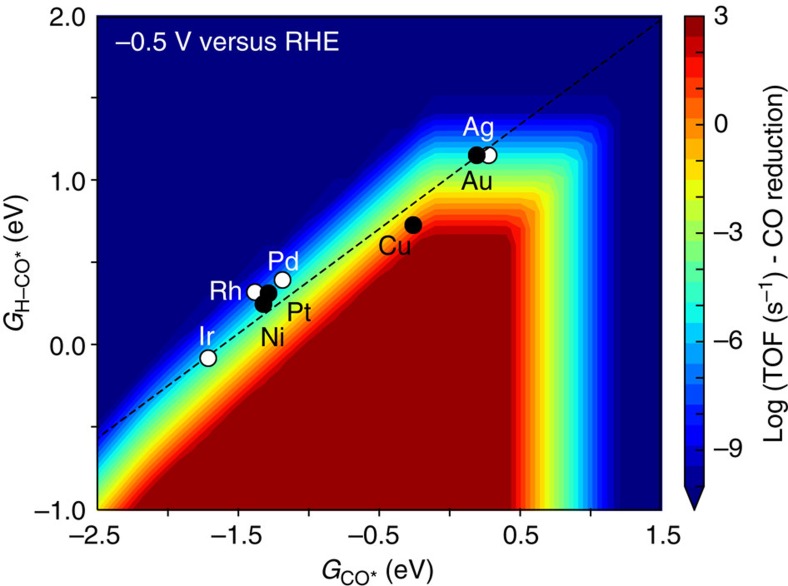
2D map of the rate of CO reduction as a function of H–CO transition state energy and CO binding energy. Performed at a potential of −0.5 V versus RHE.

The other option is to find exceptions to the scaling relation between the H–CO complex and CO in Fig. 2a. As indicated in Fig. 5, the rate could be substantially larger if we could find catalysts where the transition state is stabilized for a given CO adsorption energy.

Previously, a purely thermochemical analysis had suggested that the free energy of adsorbed CHO could be used as a simple measure of the activity^33^. On pure transition metal surfaces the H–CO complex does scale with *CHO binding energy (Fig. 2b). However, while (doped) MoS_2_ stabilizes the CHO binding energies relative to the transition metal scaling relation^28^, their corresponding transition states are not stabilized, as shown in Fig. 2b. CHO binds to a different site than CO on the sulfides, which decouples the scaling between the two energies. Unfortunately, as shown in Supplementary Fig. 7, the transition state is initial state like, and therefore this effect cannot be exploited for the transition state energy. We therefore suggest that the CHO adsorption energy can only be used as a descriptor for situations where there is no site change during the process, and that any screening study should begin with the evaluation of the energy of the H–CO transition state complex. This then poses a considerably more stringent design criterion for active catalysts than can be derived from a simple thermodynamic analysis.

In conclusion, we have presented a kinetic model for electrochemical CO reduction, based on *ab initio*, explicit solvent calculations of the energetics of the elementary steps. This kinetic model gave theoretical polarization curves, kinetic activity volcano, and selectivities on a range of transition and noble metals. We showed that the CO reduction activity is dominated by step sites, and that the activity is limited by the scaling relation between the transition state for CO hydrogenation and CO binding energies. The latter insight points to a considerably more stringent design criterion for more active catalysts for CO/CO_2_ electroreduction than a simple thermochemical analysis. Future work will focus on refinements of the model to investigate effects of C–C coupling, CO dissociation in the case of strongly binding metals, pH and mass transport, and solvation and electrolyte structures.

## Methods

### Computational details

Reaction energetics were calculated with DFT with a periodic plane-wave implementation and ultrasoft pseudopotentials using the QUANTUM ESPRESSO code^52^, interfaced with the Atomistic Simulation Environment (ASE)^53^. We applied the BEEF-vdW functional, which provides a reasonable description of van der Waals forces while maintaining an accurate prediction of chemisorption energies^32^. Plane-wave and density cutoffs were 500 and 5,000 eV, respectively, with a Fermi-level smearing width of 0.1 eV.

Convergence tests for the adsorption energies of *CO and *CHO were performed with respect to the layer thickness and number of fixed layers, with variations within 0.02 eV amongst 3–6 layer slabs. Thus, adsorption energies were evaluated using four-layer 3 × 3 supercells with the bottom two layers constrained, and (4 × 4 × 1) Monkhorst-Pack *k*-point grids^54^ were used. All structures were optimized until force components were <0.05 eV Å^−1^. A dipole correction^55^ was applied to decouple the electrostatic interaction between the periodically repeated slabs. Solvation corrections for *CO (0.2 eV) and *CHO (0.3 eV) were applied on the basis of explicit solvent calculations for Ag, Au, Cu, Pd, and Pt.

The computational hydrogen electrode^37^ was used to determine the reaction energies as a function of potential. At *U*=0 V versus RHE, protons and electrons are at equilibrium with H_2_ at 101325 Pa, 298 K, and all pH values:


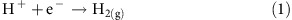


At a given *U*≠0 V versus RHE,


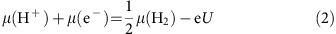


Therefore, the free energy change of, for example, the proton–electron transfer to *CO can be calculated through:


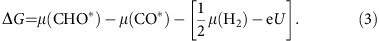


Surface thermochemical hydrogenation barriers on (211) facets were calculated using (3 × 2) supercells with Monkhorst-Pack^54^ *k*-point grids of [4 × 4 × 1]. The transition state geometry was determined through the fixed bond length method^56^. The bond length was varied by 0.01 Å per step and the forces were converged to <0.05 eV Å^−1^. The hydrogenation barriers on the (111) facets were obtained from ref. 57.

Electrochemical barriers on (111) transition metal facets were evaluated using (3 × 2), (3 × 4), (3 × 6) and (6 × 4) supercells with Monkhorst-Pack^54^ *k*-point grids of [4 × 6 × 1], [4 × 3 × 1], [4 × 2 × 1] and [2 × 3 × 1]. (100) and (211) facets were modelled with (3 × 3) supercells and Monkhorst-Pack *k*-point grids of [4 × 4 × 1]. All structures contained a three-layer transition metal slab, with atoms in the top layer relaxed and the rest fixed, along with an ice-like water structure^58^ for the (111) facets and hydrogen-bonded water layers for the (100) and (211) facets determined through minima hopping^57^,^59^. Fig. 1 shows images from the reaction pathway for proton-electron transfer to *CO on both 111 and 211 Cu facets. Excess hydrogens added to the water layer give rise to spontaneous charging of the interface, with electron transfer from hydrogen atoms to the slab^60^; this is shown in the charge density isosurfaces in Fig. 1a–c. In addition to elemental transition metal systems, we also calculated the transition state energies of CO protonation to CHO on the molybdenum edge for MoS_2_ and the sulfur edge of Ni-doped MoS_2_, modelled with a (4 × 4) S–Mo–S sandwich structure and Monkhorst-Pack *k*-point grids of [2 × 1 × 1]^61^. All systems were electroneutral and no compensating homogeneous background charge was applied. Transition state geometries and energies were calculated using the climbing-image nudged elastic band method, with the forces on the climbing-image converged to <0.05 eV Å^−1^ (ref. 33). The spring constants were tightened for images close to the saddle point^62^. The plane wave and charge density cutoff, exchange-correlation functional, and other parameters were the same as those used for geometry optimizations.

The absolute potential at the interface was determined by the work function relative to vacuum, and referenced to the experimental work function of the standard hydrogen electrode, 4.4 eV (ref. 63). GGA-level functionals can lead to incorrect band alignment of solvent and water, which leads to artificial charge transfer at the interface^64^. This problem is mitigated with the usage of counter-ions, a shift in water structure or the application of a Hubbard *U* (ref. 64). In this work, we have applied H-down water structures, which present the least issues with band alignment for negatively charged slabs. The net dipole^65^ from the H-down water orientation was found to be ∼1.3 eV for (111) surfaces and 0.8 eV for (211) surfaces, and this value was subtracted from the calculated work function to correct for the net effect of using an oriented water layer in the simulations.

The potential-dependent electrochemical kinetic barriers were obtained through the recently developed charge-extrapolation scheme^35^,^36^. All barriers were extrapolated to 4.0 eV, which corresponds to 0.0 V_RHE_ at pH=7, since experiments are performed under neutral conditions. All transition states were referenced to the initial state of aqueous protons and electrons, as determined using the computational hydrogen electrode^37^.

Charge density isosurfaces in Fig. 1a–c were calculated with the same parameter settings as for geometric optimization. The magenta and blue corresponds to an isosurface of 0.001 and –0.001 eBohr^–3^, respectively.

### Data availability

All data generated or analysed during this study are included in this published article (and its supplementary information files). See Supplementary Tables 1–3 for data in Fig. 1, Supplementary Tables 2–3 for data in Fig. 2, and Supplementary Tables 1–4 for data to reproduce Figs 3, 4, 5.

## Additional information

**How to cite this article:** Liu, X. *et al*. Understanding trends in electrochemical carbon dioxide reduction rates. *Nat. Commun.* **8,** 15438 doi: 10.1038/ncomms15438 (2017).

**Publisher's note**: Springer Nature remains neutral with regard to jurisdictional claims in published maps and institutional affiliations.

## Supplementary Material

Supplementary Information Supplementary Figures, Supplementary Tables, Supplementary Methods, Supplementary Notes and Supplementary References
