# Supplementary material for: Understanding trends in electrochemical carbon dioxide reduction rates
Source: Nat Commun. 2017 May 22;8:15438. doi: 10.1038/ncomms15438 (PMC5458145; doi:10.1038/ncomms15438)
Supplement: Supplementary Information — Supplementary Figures, Supplementary Tables, Supplementary Methods, Supplementary Notes and Supplementary References [file ncomms15438-s1.pdf]

Supplementary Note 1: Free energy corrections

For gaseous species, the electronic energies are corrected using standard ideal-gas methods. The chemical potential is given by:

$$\mu = E_{elec} + ZPE + \int C_p dT - T^*S$$

where the E_{elec} is the calculated electronic energy, ZPE the zero-point energy, C_p the heat capacity, S the entropy contribution, and T the temperature which was assumed to be 298.15K.[1,2] We applied in this work the same free energy corrections for the gaseous species as in Ref. [18]. A correction of 0.33 eV was also applied to the energy of CO_2(g), which was determined from fits to gas phase reactions in Ref. [3]. In determining the free energy diagram in Figure 1, the CO_2 pressure was assumed to be 1 bar, and differential conditions for the products. In the kinetic model, the CO pressure was assumed to be 1mbar, based on the estimated partial pressure of CO from the Faradaic efficiency under CO_2 reduction conditions[4]. The free energy of liquid water was determined using the ideal gas approximation at its vapour pressure at room temperature, 3534 Pa. For solvated protons, adsorbed intermediates, and transition states, the harmonic approximation was applied to determine the free energy corrections to the electronic energies[5]. Corrections for the adsorbates were taken from Ref. [6]. Supplementary Table 1 lists the corrections applied for reaction barriers. An overbinding correction of 0.17 eV was applied to CO* as suggested in Ref. [7].

Supplementary Note 2: Schematic of the model system

Supplementary Figure 1 shows images for the proton-electron transfer to *CO to form *CHO at the initial, transition, and final states on Cu(111) and Cu(211) surfaces.

Supplementary Note 3: Scaling relations

The adsorption and transition state energies of reaction intermediates on surfaces often scale with the adsorption energies of a small number of adsorbates (typically one or two), due to trends in the underlying surface electronic structure[8]. In this work, the adsorption energies and transition state energies scale linearly with the adsorption energies of CO* on (211) surfaces according to the following equation:

$$E_{\mathrm{ads/TS}} = \gamma \times \Delta E_{\mathrm{CO*}} + \varepsilon$$

where the parameters γ and ε are fitted for each intermediate as a function of the binding energies of CO* with all energies being relative to CO, H_2 and H_2O in the gas phase. The scaling parameters are depicted in Supplementary Figures 2 and 3.

Supplementary Note 4: Kinetics

We took a mean-field approach to microkinetic modelling, where the net rate of an elementary reaction mA ↔ nB was given by

$$r = k_+\theta_{\mathrm{A}}^{\mathrm{m}} - k_-\theta_{\mathrm{B}}^{\mathrm{n}},$$

where θ_{i} represents the surface coverage of adsorbate i, $k_{+/-}$ represents the rate constants of the forward and backward reaction respectively[9]. The rate constants were calculated through the equation $k = P\times e^{-Ga/(kbT)}$, where P represents the reaction prefactor, G_{a} represents the activation barrier, k_{b} represents the Boltzmann constant and T represents the reaction temperature. Site coverages were modeled using the pseudo-steady state approximation (i.e. the rate of change of all surface intermediate coverages is 0)[9]. These assumptions were implemented in the CatMAP software package[10], which was applied to solve the microkinetic model.

Lateral adsorbate-adsorbate interactions were modeled using a first-order expansion in the coverage for the differential adsorption energy:

$$E_i(\theta_i) = E_i^0 + \sum_j f\epsilon_{ij}\theta_j$$

where $E_i(\theta_i)$ is the differential adsorption energy of species i given a vector of coverages θ_i, E_i^0 is the differential adsorption energy of species i in the low-coverage limit, ϵ_{ij} is a matrix of interaction parameters for the interaction between species i and j, f corresponds to a piecewise-linear function for the energy as a function of coverage. The H* coverage is excluded when calculating f to account for H* being much smaller than CO and therefore has little effect on determining the strength of the interactions. Further information on the interaction model is provided in the former work[11]. The adsorbate cross-interaction parameters were determined using DFT calculations of the adsorption energies of intermediates at high coverages on Pt(111), and were listed below. Convergence of the kinetic model was achieved by first converging the solution with no interactions and incrementally increasing the interaction strength to the fitted value.

$\epsilon_{CO*,\ CO*} = 2.467$
$\epsilon_{CO*,\ H*} = 0.727$
$\epsilon_{CO*,\ H\text{-}H*} = 1.156$
$\epsilon_{CO*,\ H\text{-}ele*} = 0.786$
$\epsilon_{CO*,\ H2\text{-}ele*} = 0.509$
$\epsilon_{CO*,\ H\text{-}CO\text{-}ele*} = \epsilon_{CO*,\ H\text{-}CO\ *} = \epsilon_{CO*,\ CO\text{-}H*} = \epsilon_{CO*,\ CH\text{-}H*} = \epsilon_{CO*,\ CH2\text{-}H*} = \epsilon_{CO*,\ CH3\text{-}H*} = 1.949$
$\epsilon_{CO*,\ CO\text{-}H\text{-}ele*} = 7.867$
$\epsilon_{CO*,\ H\text{-}COH\text{-}ele*} = 1.382$
$\epsilon_{OH*,\ OH*} = 1.033$

CH*, CH_2*, CH_3*, CHOH*, CHO*, COH* were assumed to have same interactions as those of CO*. All unlisted $\epsilon_{i,\ j}$ are assumed to be zero.

Uncertainties associated with the exchange-correlation energies are provided by the BEEF-vdW ensemble. The uncertainty is propagated through the kinetic model by creating an ensemble of microkinetic models corresponding to the BEEF-vdW error estimation ensemble[12-14]. This approach ensures that correlations between energetic errors are properly accounted for. Uncertainties on relative rates were approximated by the standard deviation of relative rates and were converged with an ensemble of 500 microkinetic models.

In this simple model, CH_4(g) was taken as an example of hydrocarbon/alcohol products, and H_2(g) was included as the main side product. Accordingly the experimental polarization curves included all products further reduced from CO. Both proton-electron transfer and surface hydrogenation pathways were taken into account. The steps after forming CHO* were assumed to be barrierless as we have shown that all the successive steps have smaller barriers than CO* protonation to CHO* on Cu(211). Including the barriers of these steps did not alter the overall reaction rate. We have also shown that on Cu(211) the protonation barrier to form CHOH* from COH* is about 0.6 eV higher than that of forming CHO* from CO*. To get an upper limit of the performance of the COH pathway, we used the CO* to CHO* barrier to estimate the COH*

protonation barrier on (211) surface. COH* protonation barriers on (111) surfaces were explicitly calculated. All the elementary steps are described as follows:

1. $H^+ + e^- + * \leftrightarrow H*$
2. $H^+ + e^- + H* \leftrightarrow H_2(g) + *$
3. $H* + H* \leftrightarrow H_2(g) + 2*$
4. $OH* + H^+ + e^- \leftrightarrow H_2O(g) + *$
5. $CO(g) + * \leftrightarrow CO*$
6. $CO* + H^+ + e^- \leftrightarrow CHO*$
7. $CO* + H* \leftrightarrow CHO* + *$
8. $CHO* + H^+ + e^- \leftrightarrow CHOH*$
9. $CO* + H^+ + e^- \leftrightarrow COH*$
10. $CO* + H* \leftrightarrow COH* + *$
11. $COH* + H^+ + e^- \leftrightarrow CHOH*$
12. $CHOH* + H^+ + e^- \leftrightarrow CH* + H_2O(g)$
13. $CH* + H^+ + e^- \leftrightarrow CH_2*$
14. $CH* + H* \leftrightarrow CH_2* + *$
15. $CH_2* + H^+ + e^- \leftrightarrow CH_3*$
16. $CH_2* + H* \leftrightarrow CH_3* + *$
17. $CH_3* + H^+ + e^- \leftrightarrow CH_4(g) + *$
18. $CH_3* + H* \leftrightarrow CH_4(g) + 2*$

where * represents a surface site. All the steps had prefactors of 10^{13} based on harmonic transition state theory[9].

The potential dependence of the electrochemical steps is determined from the charge of transition state and are listed below:

dG_a(step1, 111 surface)/dU = –0.4
dG_a(step2, 111 surface)/dU = –1.0
dG_a(step1, 211 surface)/dU = –0.7
dG_a(step1, 100 surface)/dU = –0.8
All unlisted electrochemical steps use dependences of –0.5.

Supplementary Note 5: Relative Rates of CO reduction and H_2 production

In Supplementary Figure 4, the rate relative to that of Cu(211) is shown as a function of CO* binding energy, along with the relative uncertainty determined through BEEF-vdW error estimation ensembles. Cu(211) remains to be close to the volcano peak even when uncertainties are accounted for.

Supplementary Note 6: *CO and *H coverages

Overall, *CO and *H predominated under CO reduction conditions. Supplementary Figure 5 shows the coverages of these species at –0.5 and –1.0 V vs. RHE.

Supplementary Note 7: Projected density of states at the transition state

In general, processes taking place at a metal surface are not limited by electron transfer[15]. This is illustrated in Supplementary Figure 6, which shows the projected density of states for the transition state of proton-electron transfer to CO, obtained using a very small smearing width of

0.01 eV and *k*-point grid of [20×20×1]. Since the width of the adsorbate-induced states is on the order of eV, the Heisenberg uncertainty principle suggests the rate of electron jumps between the adsorbate and metal surface to be more than 10^{15} s^{-1}.

Supplementary Note 8: Transition state configurations for molybdenum sulfide and Ni-doped molybdenum sulfide

From a thermodynamic perspective, MoS_2 type materials break the scaling between *CO and *CHO, since *CO binds to metal edge sites and *CHO the sulfur ones[16]. However, such a deviation is not shown in the transition state scaling lines of Figure 2. This result can be rationalized by examining the configuration of the associated transition states, shown in Supplementary Figure 7 for MoS_2 and $NiMoS_2$. The CO→CHO transition state is initial state like, i.e. bound to the metal site, and so the stabilization of *CHO by the sulfur site does not affect the transition state.

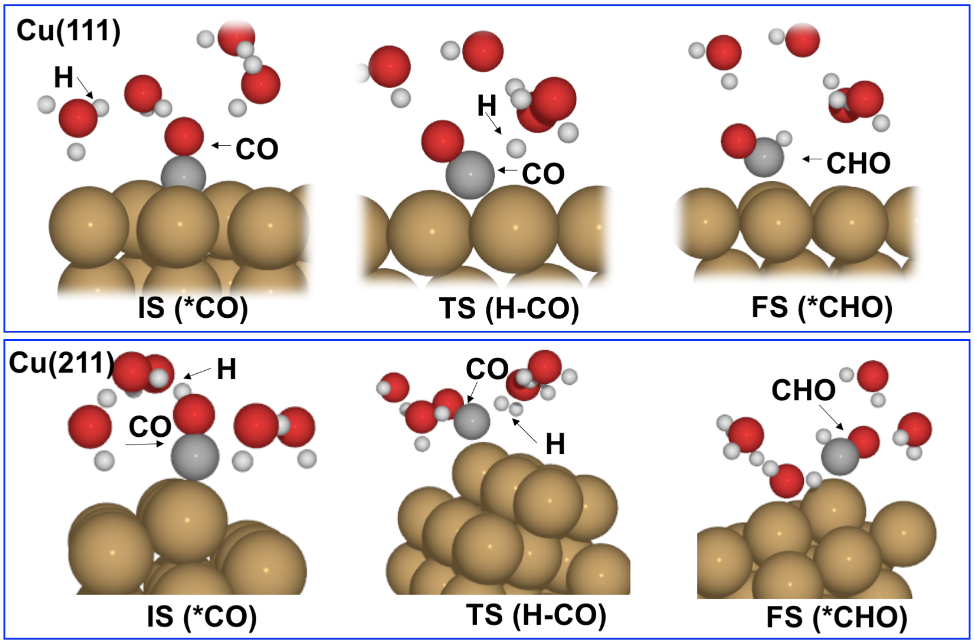

Supplementary Figure 1 Images along the reaction pathway for the proton-electron transfer to *CO to form *CHO on Cu (111) and (211) facets.

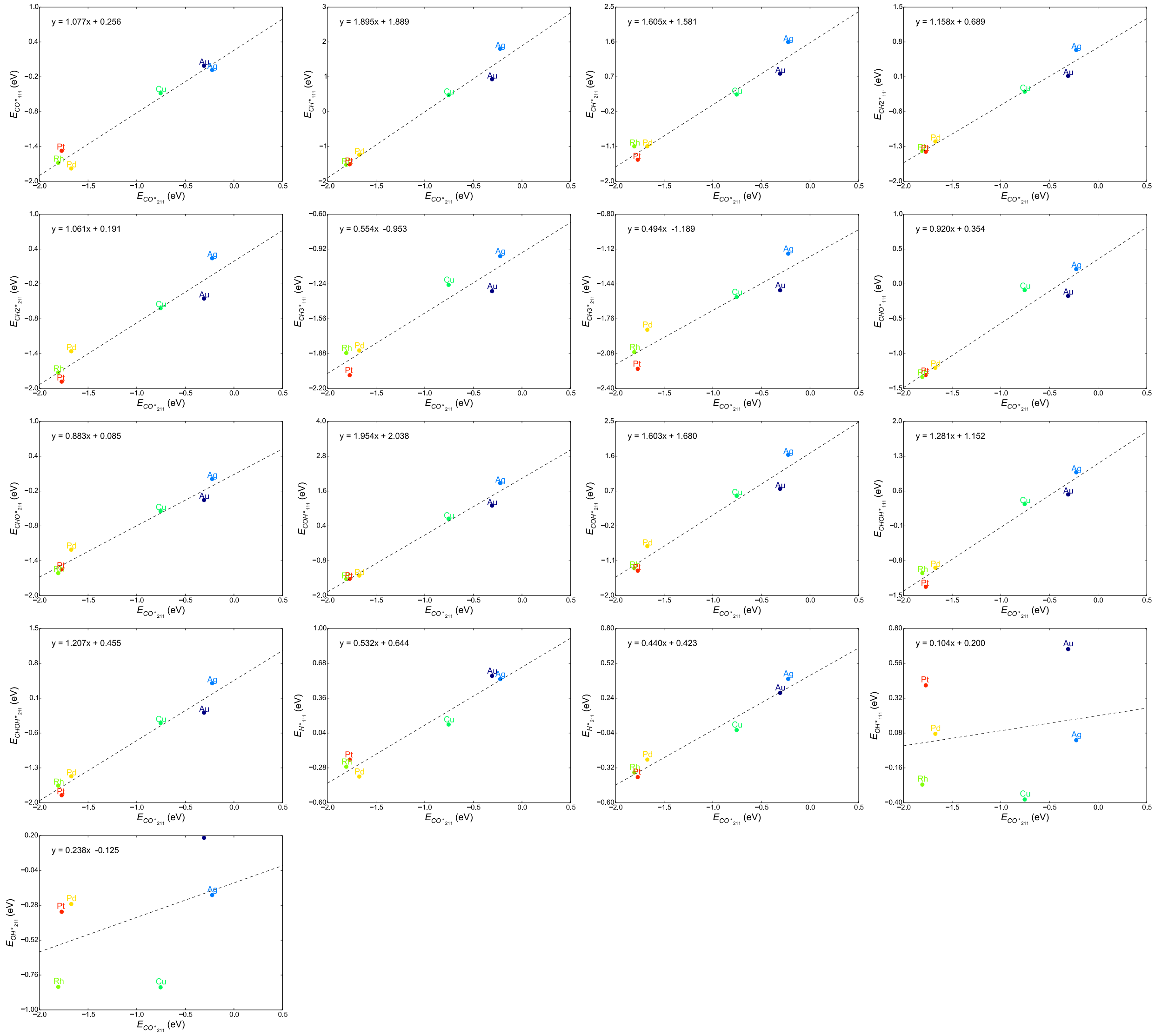

Supplementary Figure 2 Adsorbate scaling relations

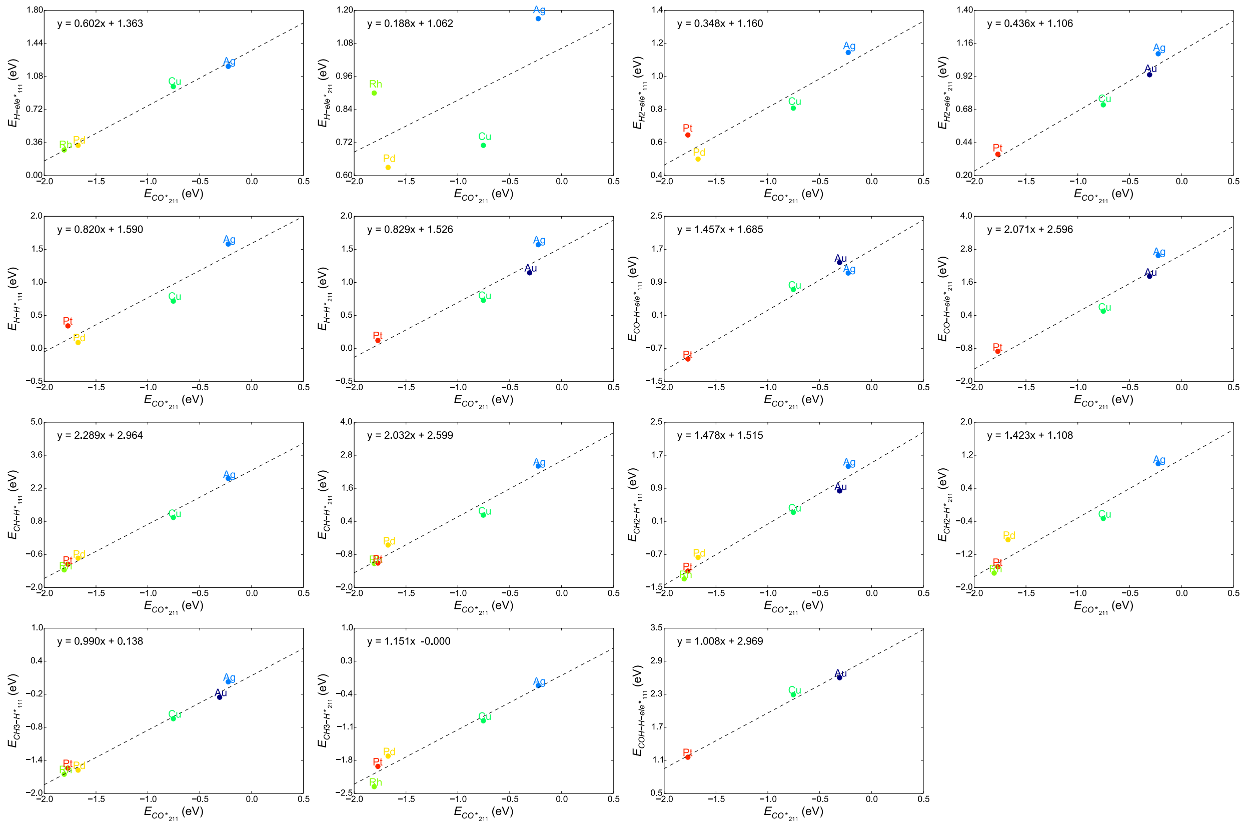

Supplementary Figure 3 Transition state scaling relations

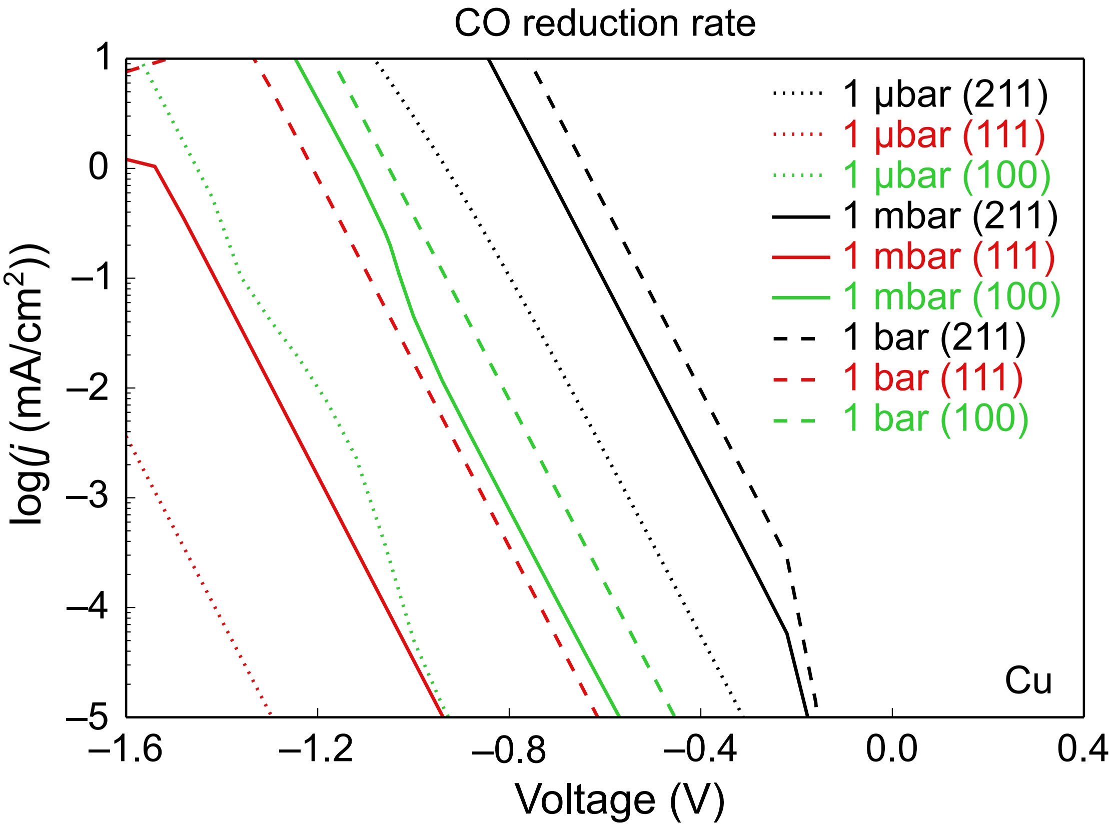

Supplementary Figure 4 Pressure dependence on Cu(100), Cu(111) and Cu(211).

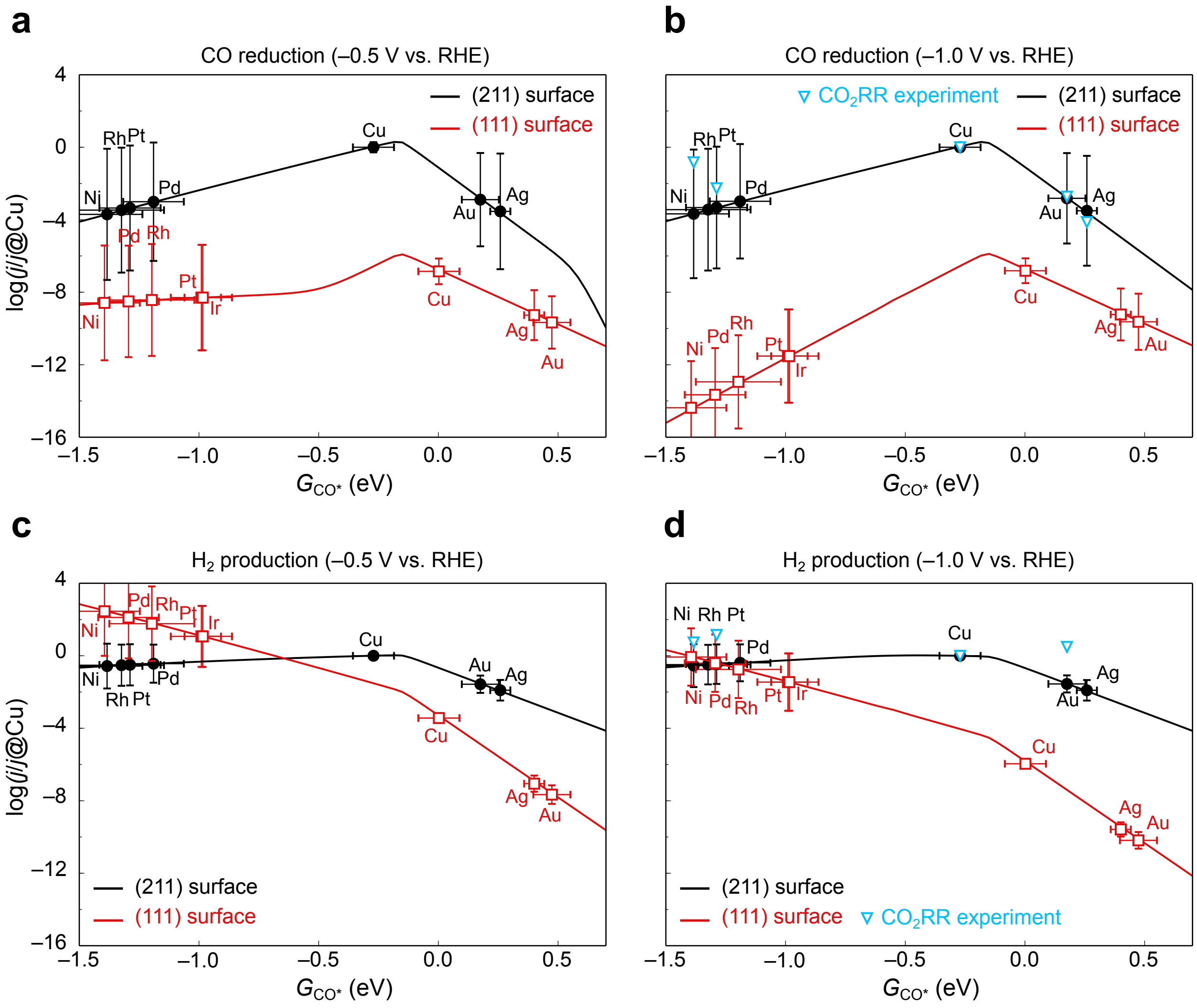

Supplementary Figure 5 Relative CO reduction and hydrogen evolution volcanoes as a function of CO binding energy, as determined through microkinetic modeling. Cu(211) was taken as the reference.

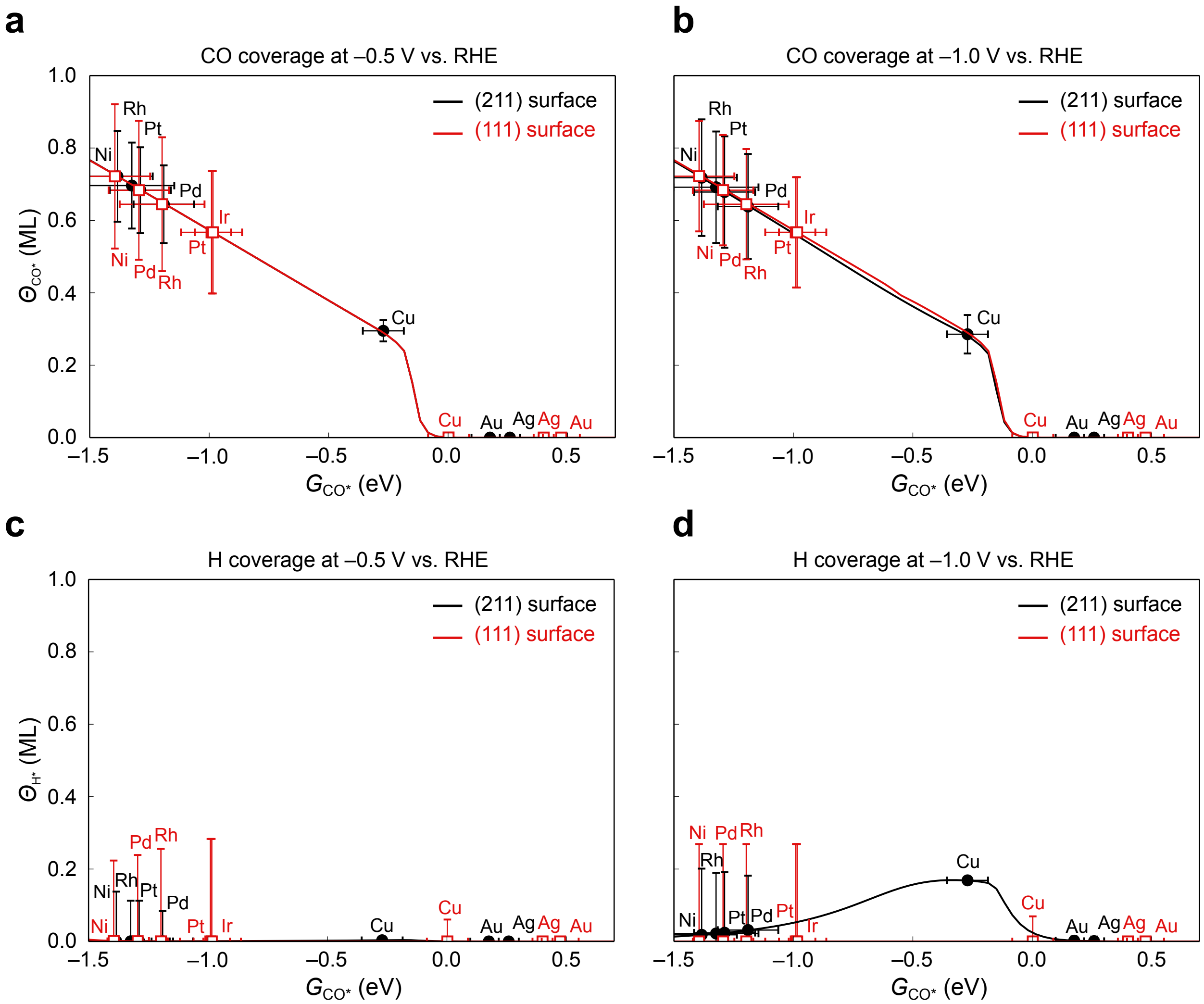

Supplementary Figure 6 Coverages of CO and H at –0.5 and –1.0 V_{RHE}

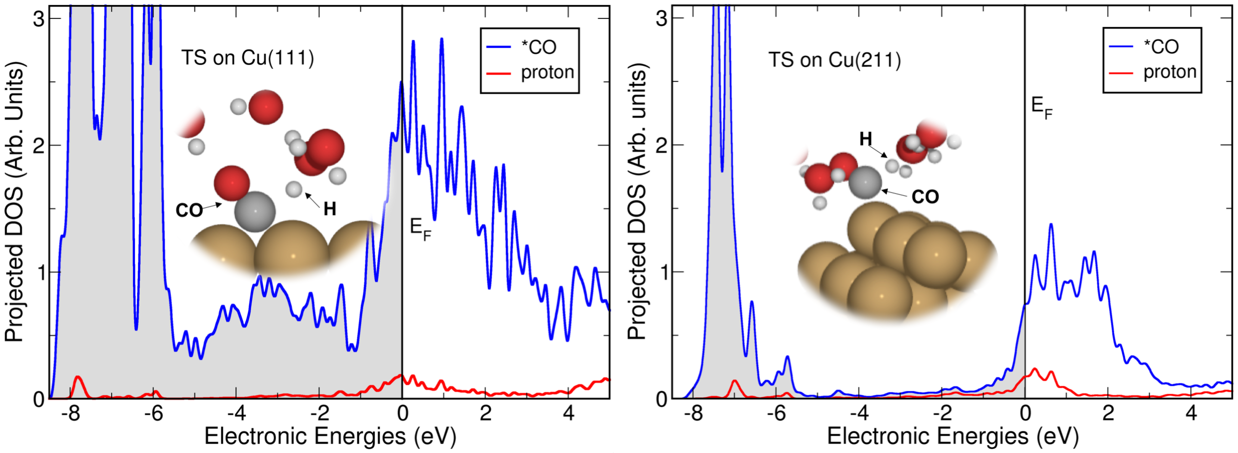

Supplementary Figure 7 Calculated projected density of states (PDOS) of the transition state of CO protonation to CHO on Cu(111) and Cu(211) surfaces.

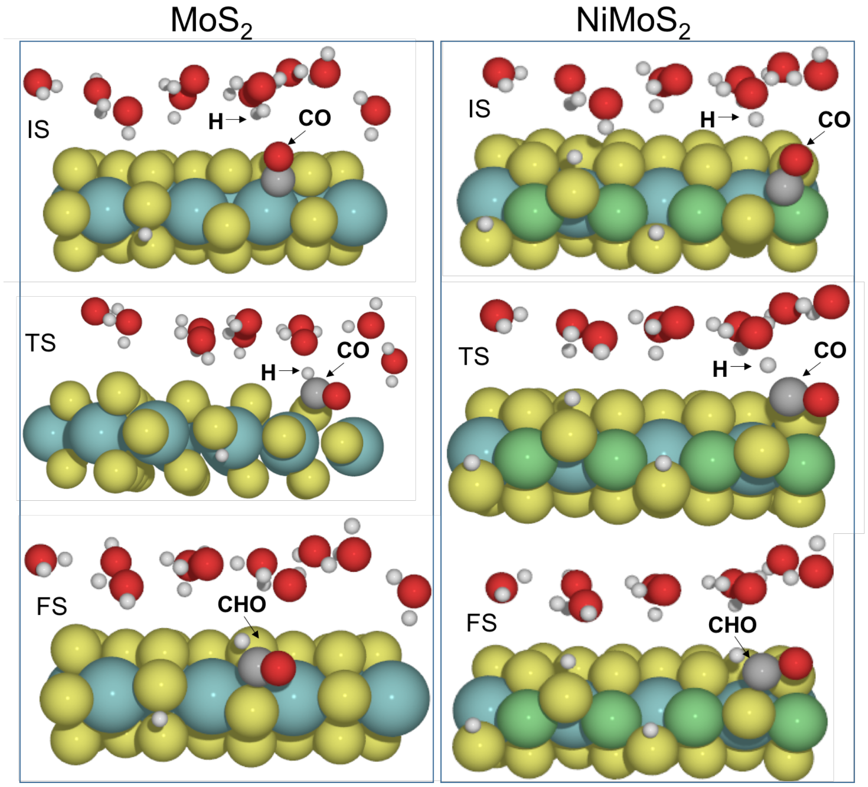

Supplementary Figure 8 The transition states images of CO protonation to CHO on the edges of MoS_2 and Ni-doped MoS_2.

Supplementary Table 1 Free energy corrections to the electronic energies applied to the various reaction barriers considered. Transition state energies are corrected by their zero-point energy.

Reactions	Surfaces	*CO (eV)	H-CO (eV)	ΔG_a (eV)
$* CO + H^+ + e^- \rightarrow * CHO$	Cu(111)	0.19	0.34	0.15
	Pt(111)	0.19	0.42	0.23
	Au(111)	0.19	0.38	0.19
	Ag(111)	0.17	0.44	0.27
	Cu(211)	0.19	0.34	0.15
	Pt(211)	0.20	0.37	0.17
	Au(211)	0.19	0.34	0.15
	Ag(211)	0.17	0.34	0.17
	Pd(211)	0.17	0.34	0.17
	MoS_2	0.24	0.43	0.19
	$NiMoS_2$	0.24	0.43	0.19
$* CO + H^+ + e^- \rightarrow * COH$	Cu(111)	0.19	0.43	0.24
	Pt(111)	0.19	0.53	0.34
	Au(111)	0.19	0.45	0.26
	Ag(111)	0.17	0.40	0.23
	Cu(211)	0.19	0.43	0.24
	Pt(211)	0.20	0.53	0.33
	Au(211)	0.19	0.49	0.30
	Ag(211)	0.17	0.44	0.27
$* CO_2 + H^+ + e^- \rightarrow * COOH$	Cu(211)	0.32	0.62	0.30
$* COOH + H^+ + e^- \rightarrow * CO + H_2O$	Cu(211)	0.62	0.76	0.14
$* CHOH + H^+ + e^- \rightarrow * CH + H_2O$	Cu(211)	0.71	0.92	0.21
$* CH + H^+ + e^- \rightarrow * CH_2$	Cu(211)	0.35	0.58	0.23
$* CH_2 + H^+ + e^- \rightarrow * CH_3$	Cu(211)	0.63	0.81	0.18
$* CH_3 + H^+ + e^- \rightarrow CH_4$	Cu(211)	0.93	1.12	0.19
$* CHO + H^+ + e^- \rightarrow * CHOH$	Cu(211)	0.34	0.71	0.37
$* CHO + H^+ + e^- \rightarrow * CHOH$	Cu(111)	0.46	0.71	0.25
$* COH + H^+ + e^- \rightarrow * CHOH$	Cu(211)	0.19	0.34	0.15
$* COH + H^+ + e^- \rightarrow * CHOH$	Cu(111)	0.19	0.34	0.15

Supplementary Table 2 Barriers from Figure 1 and 2 in eV

Figure 1	
$CO_2 + (e^- + H^+) \rightarrow COOH$	0.21
$COOH + (e^- + H^+) \rightarrow (CO{+}H_2O)$	0.67
$CO + (e^- + H^+) \rightarrow CHO$	0.97
$CHO + (e^- + H^+) \rightarrow CHOH$	0.78
$CHOH + (e^- + H^+) \rightarrow (CH + H_2O)$	0.69
$CH + (e^- + H^+) \rightarrow CH_2$	0.37
$CH_2 + (e^- + H^+) \rightarrow CH_3$	0.29
$CH_3 + (e^- + H^+) \rightarrow CH_4$	0.55
$CO + (e^- + H^+) \rightarrow COH$	1.31
$COH + (e^- + H^+) \rightarrow CHOH$	1.62
Figure 2	
$CO + (e^- + H^+) \rightarrow$ CHO on Ag211	0.90
$CO + (e^- + H^+) \rightarrow$ CHO on Au211	0.95
$CO + (e^- + H^+) \rightarrow$ CHO on Pt211	1.58
$CO + (e^- + H^+) \rightarrow$ CHO on Pd211	1.56
$CO + (e^- + H^+) \rightarrow$ CHO on Ir211	1.46
$CO + (e^- + H^+) \rightarrow$ CHO on Pt211-2/3CO ML	1.51
$CO + (e^- + H^+) \rightarrow$ CHO on Pt211-3/3CO ML	1.17
$CO + (e^- + H^+) \rightarrow$ CHO on MoS_2	0.88
$CO + (e^- + H^+) \rightarrow$ CHO on $NiMoS_2$	1.35
$CO + (e^- + H^+) \rightarrow$ CHO on Ag111	1.05
$CO + (e^- + H^+) \rightarrow$ CHO on Au111	0.98
$CO + (e^- + H^+) \rightarrow$ CHO on Cu111	1.34
$CO + (e^- + H^+) \rightarrow$ CHO on Pt111-1/6CO ML	1.89
$CO + (e^- + H^+) \rightarrow$ CHO on Pt111-2/6CO ML	1.63
$CO + (e^- + H^+) \rightarrow$ CHO on Pt111-3/6CO ML	1.54
$CO + (e^- + H^+) \rightarrow$ CHO on Cu100	1.26

Supplementary Table 3 Adsorption energies of all reaction intermediates used in kinetics, in eV

Surfaces	Eads (eV)								
	H*	CO*	CHO*	COH*	CHOH*	CH*	CH_2*	CH_3*	OH*	COOH*
Ag(211)	0.40	-0.22	0.01	1.64	0.40	1.60	0.24	-1.16	-0.21	/
Au(211)	0.28	-0.31	-0.35	0.76	-0.19	0.78	-0.45	-1.50	0.18	/
Cu(211)	-0.02	-0.75	-0.55	0.58	-0.40	0.24	-0.62	-1.56	-0.84	-0.87
Pd(211)	-0.25	-1.67	-1.21	-0.72	-1.47	-1.09	-1.36	-1.86	-0.27	/
Pt(211)	-0.39	-1.77	-1.55	-1.36	-1.85	-1.44	-1.88	-2.22	-0.32	/
Rh(211)	-0.36	-1.81	-1.61	-1.29	-1.66	-1.09	-1.72	-2.07	-0.84	/
Ni(211)	-1.53	-1.87	-1.56	/	-1.65	-1.55	-1.69	-2.04	-1.19	/
Ir(211)	-0.57	-2.19	-1.93	-1.75	-1.95	-1.80	-2.16	-2.40	-0.91	/
Ag(111)	0.54	-0.08	0.21	1.87	0.98	1.81	0.64	-0.99	0.03	/
Au(111)	0.56	-0.01	-0.17	1.10	0.53	0.93	0.12	-1.31	0.66	/
Cu(111)	0.12	-0.48	-0.09	0.64	0.34	0.47	-0.20	-1.25	-0.38	/
Pd(111)	-0.36	-1.78	-1.20	-1.31	-0.94	-1.24	-1.20	-1.85	0.07	/
Pt(111)	-0.20	-1.47	-1.31	-1.42	-1.32	-1.51	-1.40	-2.08	0.41	/
Rh(111)	-0.27	-1.68	-1.33	-1.43	-1.05	-1.52	-1.39	-1.87	-0.27	/
Ni(111)	-0.47	-1.88	-1.33	-1.55	-1.19	-1.57	-1.56	-1.83	-0.49	/
Ir(111)	-0.19	-1.47	-1.14	-1.56	-1.21	-1.70	-1.49	-1.99	-0.02	/
Cu(100)	0.11	-0.62	-0.25	0.12	0.01	-0.34	-0.42	-1.46	-0.59	/

Supplementary Table 4 Transition state energies of all chemical steps used in kinetics in eV

Surfaces	ETS (eV)				
	H-CO*	CO-H*	CH-H*	CH2-H*	CH3-H*
Ag(211)	0.82	/	2.41	1.00	-0.22
Cu(211)	0.21	1.69	0.63	-0.33	-0.96
Pd(211)	-0.65	-0.08	-0.46	-0.84	-1.71
Pt(211)	-0.63	-0.80	-1.11	-1.50	-1.93
Rh(211)	-0.01	0.08	-1.13	-1.65	-2.36

Ag(111)	0.97	2.75	2.62	1.43	0.03
Au(111)	/	/	/	0.84	-0.25
Cu(111)	0.36	1.63	0.97	0.32	-0.64
Pd(111)	-0.50	0.05	-0.76	-0.77	-1.58
Pt(111)	-0.36	-0.02	-1.01	-1.10	-1.54
Rh(111)	-0.60	-0.11	-1.25	-1.29	-1.65

References

1. Stoyanov, E.S., Stoyanova, I.V. & Reed, C.A. The structure of the hydrogen ion (H_{aq}^{+}) in Water. *J. Am. Chem. Soc.* **132,** 1484-1485 (2010).
2. Huan, T.N. *et al.* From molecular copper complexes to composite electrocatalytic materials for selective reduction of CO_2 to formic acid. *J. Mater. Chem. A* **3,** 3901-3907 (2015).
3. Studt, F., Abild-Pedersen, F., Varley, J.B. & Nørskov, J.K. CO and CO_2 hydrogenation to methanol calculated using the BEEF-vdW functional. *Catal. Lett.* **143,** 71-73 (2012).
4. Studt, F., Sharafutdinov, I. & Abild-Pedersen, F. Discovery of a Ni-Ga catalyst for carbon dioxide reduction to methanol. *Nat. Chem.* **6,** 320-324 (2014).
5. Cramer, C.J. *Essentials of Computational Chemistry: Theories and Models*, (Wiley, 2013).
6. Shi, C., Chan, K. & Norskov, J. Barriers of electrochemical CO_2 reduction on transition metals. *Org. Process Res. Dev.* **20,** 1424-1430 (2016).
7. Abild-Pedersen, F. & Andersson, M.P. CO adsorption energies on metals with correction for high coordination adsorption sites - A density functional study. *Surf. Sci.* **601,** 1747-1753 (2007).
8. Abild-Pedersen, F. Computational catalyst screening: Scaling, bond-order and catalysis. *Catal. Today* **272,** 6-13 (2016).
9. Norskov, J.K., Studt, F., AbildPedersen, F. & Bligaard, T. *Fundamental Concepts in Heterogeneous Catalysis* (Wiley, 2014).
10. Medford, A.J. *et al.* CatMAP: A software package for descriptor-based microkinetic mapping of catalytic trends. *Catal. Lett.* **145,** 794-807 (2015).
11. Kaneco, S. *et al.* Electrochemical reduction of carbon dioxide to ethylene with high Faradaic efficiency at a Cu electrode in CsOH/methanol. *Electrochim. Acta* **44,** 4701-4706 (1999).
12. Medford, A.J. *et al.* Assessing the reliability of calculated catalytic ammonia synthesis rates. *Science* **345,** 197-200 (2014).
13. Yang, N. *et al.* Intrinsic selectivity and structure sensitivity of rhodium catalysts for C_{2+} oxygenate production. *J. Am. Chem. Soc.* **138,** 3705-3714 (2016).
14. Wellendorff, J. *et al.* Density functionals for surface science: Exchange-correlation model development with Bayesian error estimation. *Phys. Rev. B* **85,** 235149-23 (2012).
15. Kwon, Y. & Lee, J. Formic acid from carbon dioxide on nanolayered electrocatalyst. *Electrocatalysis* **1,** 108-115 (2010).

16. Hong, X., Chan, K., Tsai, C. & Nørskov, J.K. How doped MoS_2 breaks transition-metal scaling relations for CO_2 electrochemical reduction. *ACS Catal.* **6,** 4428-4437 (2016).
